# Supplementary material for: Cellular stressors contribute to the expansion of hematopoietic clones of varying leukemic potential
Source: Nat Commun. 2018 Jan 31;9:455. doi: 10.1038/s41467-018-02858-0 (PMC5792556; doi:10.1038/s41467-018-02858-0)
Supplement: Supplementary file 1 — Supplementary Information [file 41467_2018_2858_MOESM1_ESM.pdf]

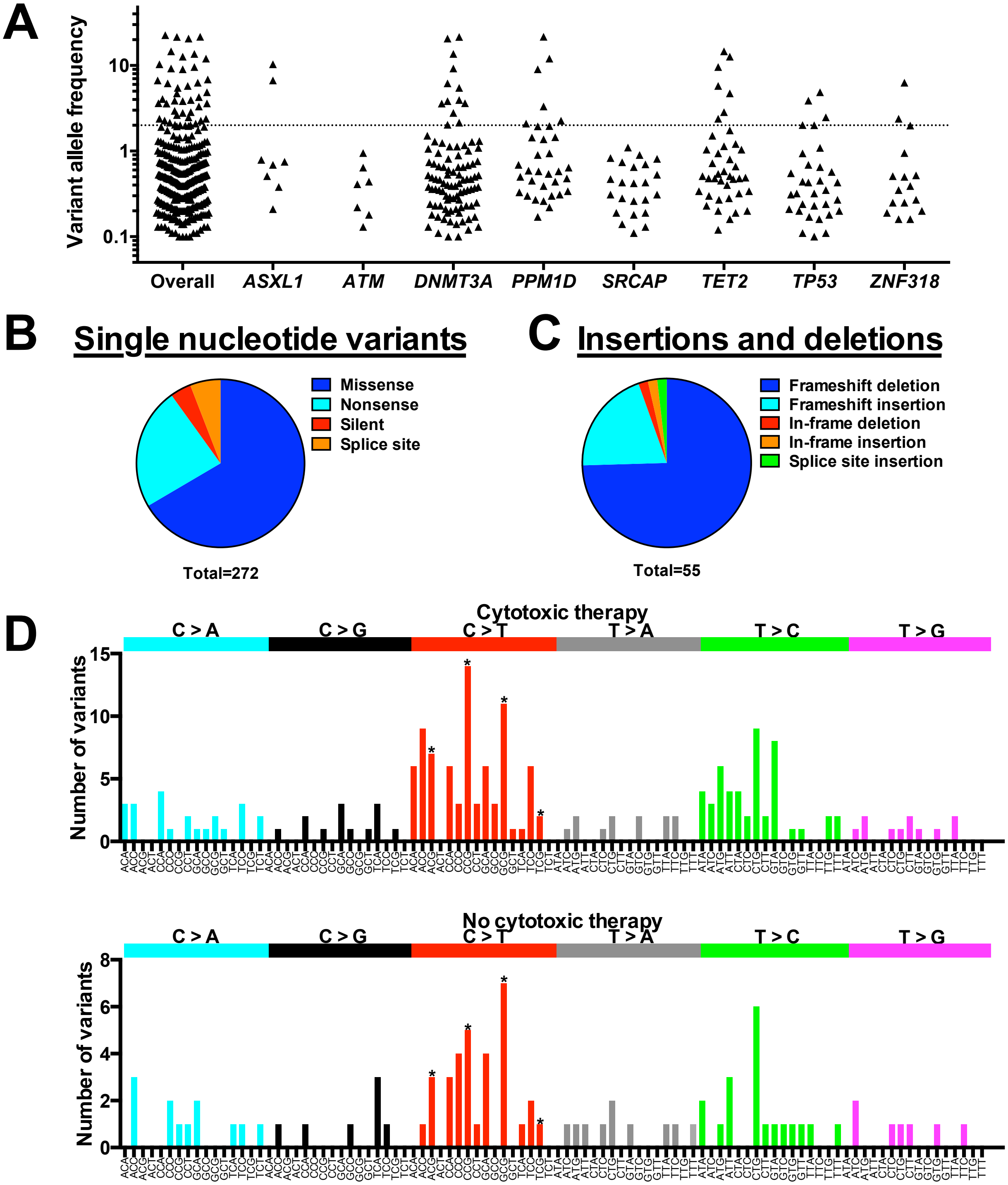


**Supplementary Figure 1. Error-corrected HaloPlex sequencing allows for the detection of somatic variants at increased sensitivity. a,** VAF distribution of the 327 variants identified by Haloplex error-corrected sequencing in 138 pheresis specimens and the VAF distribution of those variants in commonly mutated genes. **b,** Characterization of the 272 SNVs detected in pheresis samples by HaloPlex error-corrected sequencing. **c,** Characterization of the 55 indels detected in pheresis samples by HaloPlex error-corrected sequencing. **d,** Trinucleotide context in which the SNVs identified in pheresis samples occurred either after exposure to cytotoxic therapy (n=185) or in the absence of cytotoxic therapy exposure (n=87). * The mutation is a C to T transition mutation occurring in the context of a CpG.

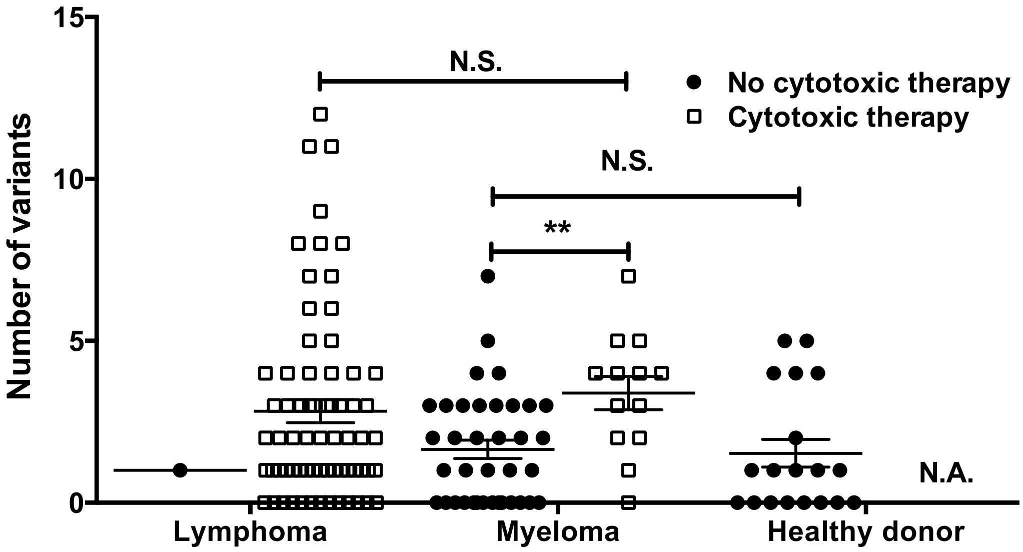


**Supplementary Figure 2. Cytotoxic therapy exposure is associated with clonal hematopoiesis.** The total number of clonal hematopoiesis-associated variants identified in lymphoma patients (n=69), myeloma patients (n=50), and healthy donors (n=19) grouped by whether or not these individuals previously received cytotoxic therapy. Myeloma patients receiving cytotoxic therapy (n=13) had significantly more variants identified than myeloma patients not receiving cytotoxic therapy (n=37) and were not statistically different from lymphoma patients receiving cytotoxic therapy (n=68). In contrast, myeloma patients not receiving cytotoxic therapy were not statistically different from healthy donors. N.S. Not significant. N.A. Not applicable. ** $P<0.01$. Significance was determined with a negative-binomial regression analysis. Data is represented as the mean ± the standard error of the mean.

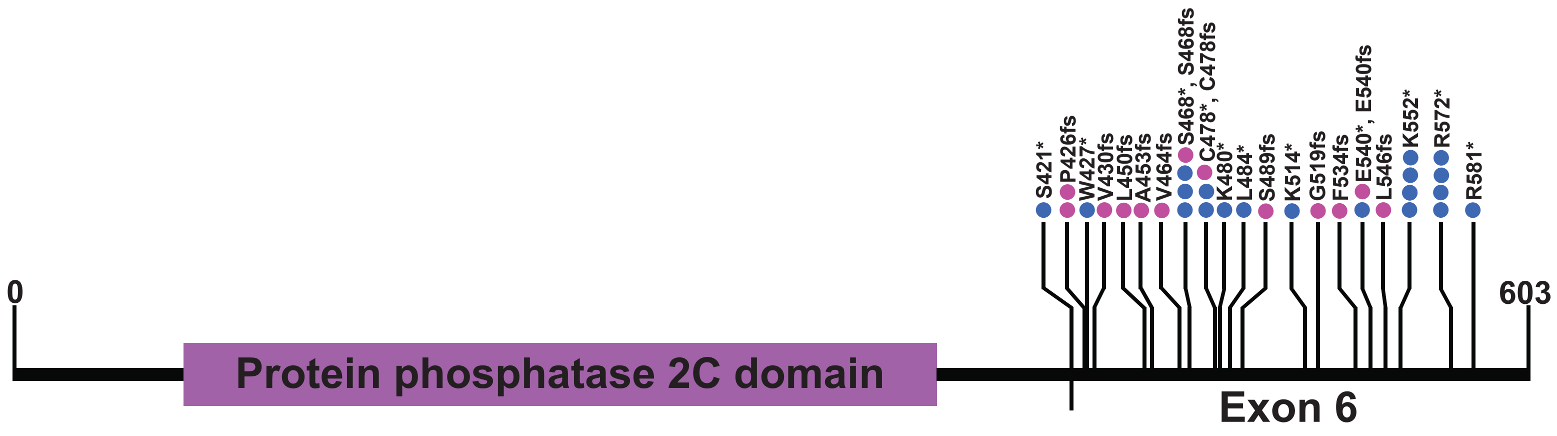


**Supplementary Figure 3. Lollipop plot of *PPM1D* mutations.** 33 *PPM1D* SNVs and indels were identified by HaloPlex sequencing in 138 pheresis specimens. Blue circle: nonsense mutation. Purple circle: frameshift mutation.

## Cytotoxic therapy exposure

| Patient | 746 | 26 | 34 | 925 | 653 | 815 | 753 | 973 | 643 | 31 | 484 | 775 | 725 | 737 | 731 | 120 | 601 | 599 | 644 | 459 |
|---|---|---|---|---|---|---|---|---|---|---|---|---|---|---|---|---|---|---|---|---|
| Age | 61 | 62 | 53 | 68 | 59 | 61 | 56 | 56 | 54 | 66 | 63 | 72 | 69 | 64 | 54 | 36 | 72 | 76 | 52 | 63 |
| *PPM1D* | 6 | 4 | 4 | 2 | 1 | 2 | 2 | 2 | 2 | 1 | 1 | 1 | 1 | 1 | 1 | 1 | | | | |
| *TP53* | 1 | 2 | 1 | 2 | 1 | | | | | 1 | | | | | | | 4 | 3 | 3 | 2 |
| *ATM* | | 1 | | | | 1 | 1 | | | | 1 | | | | | | | | | |
| *SRCAP* | | | 1 | | 1 | | | | | 1 | | 2 | | | | | | | 1 | |
| *BRCC3* | | | | | | | | | | | | | | | | | | | | 2 |
| *DNMT3A* | | 1 | 3 | | 7 | 2 | | 1 | | | | 1 | 1 | | | | | | | 2 |
| *TET2* | 1 | | 1 | 1 | | | | | | | | 1 | | | 1 | | | | | |
| *ZNF318* | | | 1 | | | 1 | | | | | | | | | | | | | | |
| *ASXL1* | | | | | 1 | | | | | | | | | | 1 | | | | | |
| *BCORL1* | | | | | | | | | | | | | | | | | 1 | | | 1 |
| *GNAS* | 1 | | | | | | | | | | | | | | | | | | | |
| *SMC1A* | | | | | | | | | | | | 1 | | | | | | | | |
| *STAT3* | | | | | | | | | | 1 | | | | | | | | | | |
| *CUX1* | | | | 1 | | | | | | | | 1 | | | | | | | | |
| *CBL* | | | | | | | | | | | | 1 | | | | | | | | |
| *KRAS* | | | | | | | | | | | | | | | | | | | | |
| *BCOR* | 2 | | | | | | | | | | 1 | | | | | | | | | |
| *EZH2* | | | | | | | | | | | | | 1 | | | | | | | |
| *GNB1* | | | | | | | | | | | | | | | | | | | | |
| *SMC3* | | | | | | | | | | | | | | | | | | | | |
| *FLT3* | | | | | 1 | | | | | | | | | | | | | | | |
| *CSF3R* | | | | | | | | | | | | | 1 | | | | | | | |
| *IDH2* | | | | | | | | | | | | | | | 1 | | | | | |
| *ZRSR2* | | | | | | | | | | | | | | 1 | | | | | | |
| *SF1* | | | | | | | | | | | | | | | | | | | | |
| *JAK2* | | | | | | | | | | 1 | | | | | | | | | | |
| *SETD2* | | | | | | 1 | | | | | | | | | | | | | | |
| *RUNX1* | | | | | | | | | | | | | | | | | | | | |
| *SRSF2* | | | | | | | | | | | | | | | | | | | | |
| *ZBTB33* | | | | | | | | | | | | | | | | | | | | |
| *CREBBP* | | | | | | | | | | | | | | | | | | | | |
| *KDM6A* | | | | | | | | | | | | | | | | | | | | |
| *MYD88* | | | | | | | | | | | | | | | | | | | | |
| *PHF6* | | | | | | | | | | | | | | | | | | | | |
| *PTPN11* | | | | | | | | | | | | | | | | | | | | |
| *STAG2* | | | | | | | | | | | | | | | | | | | | |
| *SETBP1* | | | | | | | | | | | | | | | | | | | | |
| Total | 11 | 8 | 11 | 6 | 12 | 7 | 3 | 3 | 2 | 5 | 3 | 8 | 4 | 2 | 4 | 1 | 5 | 3 | 4 | 7 |

| Patient | 94 | 45 | 987 | 823 | 739 | 27 | 274 | 178 | 141 | 585 | 570 | 505 | 938 | 608 | 986 | 907 | 968 | 648 | 457 | 135 |
|---|---|---|---|---|---|---|---|---|---|---|---|---|---|---|---|---|---|---|---|---|
| Age | 58 | 70 | 72 | 58 | 53 | 57 | 64 | 69 | 43 | 69 | 59 | 64 | 54 | 70 | 23 | 41 | 55 | 49 | 68 | 63 |
| *PPM1D* | | | | | | | | | | | | | | | | | | | | |
| *TP53* | 1 | 1 | 1 | 1 | 1 | 1 | 1 | | | | | | | | | | | | | |
| *ATM* | | | | | | | | 2 | 1 | | | | | | | | | | | |
| *SRCAP* | 1 | | | | | 1 | | | | 5 | 2 | 1 | 1 | 1 | 1 | | | | | |
| *BRCC3* | | | | | | | | | | | | | | | | 1 | 1 | 1 | | |
| *DNMT3A* | | 4 | 1 | 1 | | | | 1 | 1 | 3 | 2 | 1 | | | | 2 | 2 | | 3 | 3 |
| *TET2* | 1 | 1 | | | 1 | | | | | | | | | | | | | | | |
| *ZNF318* | | 2 | 1 | | | 1 | | | | 1 | | 1 | | | | | | | 1 | 1 |
| *ASXL1* | | | | | | | | | | | | | | | | 1 | | | 1 | |
| *BCORL1* | | | 1 | | | | | | | | | | | | | | | | | |
| *GNAS* | | | | | | | | | | | | | | | | | | | | |
| *SMC1A* | | | | | | | | | | | | | | | | | | | | |
| *STAT3* | | | | | | | | | | | | | | | | | | | | |
| *CUX1* | | | | | 1 | | | | | | | | | | | | | | | |
| *CBL* | | | | | | | | | | | | | | | | | | | | |
| *KRAS* | | | | | | | | | | | | | | | | | | | | |
| *BCOR* | | | | | | | | | | | | | 1 | | | | | | | |
| *EZH2* | | | | 1 | | | | | | | | | | | | | | | | 1 |
| *GNB1* | | | | | | | | | | | | | | | | | | | | 1 |
| *SMC3* | | | | | | 1 | | | | | | | | 1 | | | | | | |
| *FLT3* | | | | | | | | | | | | | | | | | | | | |
| *CSF3R* | | | | | | | | | | | | | | | | | | | | |
| *IDH2* | | | | | | | | | | | | | | | | | | | | |
| *ZRSR2* | | | | | | | | | | | | | | | | | | | | |
| *SF1* | | | | | | 1 | | | | | | | | | | | | | | |
| *JAK2* | | | | | | | | | | | | | | | | | | | | |
| *SETD2* | | | | | | | | | | | | | | | | | | | | |
| *RUNX1* | | | | 1 | | | | | | | | | | | | | | | | |
| *SRSF2* | | | | | | | | | | | | | | | | | | | | |
| *ZBTB33* | | | | | | | | | | | | | 1 | | | | | | | |
| *CREBBP* | | | | | | | | | | | | | | | | | | | 2 | |
| *KDM6A* | | | | | | | | | | | | | | | | | | | | |
| *MYD88* | | | | | | | | | | | | | | | | | | | | |
| *PHF6* | | | | | | | | | | | | | | | | | | | | |
| *PTPN11* | | | | | | | | | | | | | | | | | | | | |
| *STAG2* | | | | | | | | | | | | | | | | | | | | |
| *SETBP1* | | | | | | | | | | | | | | | | | | | | |
| Total | 3 | 8 | 4 | 4 | 3 | 5 | 1 | 3 | 2 | 9 | 4 | 3 | 3 | 2 | 1 | 4 | 3 | 1 | 7 | 6 |

| Patient | 954 | 915 | 739 | 650 | 355 | 926 | 423 | 170 | 218 | 260 | 368 | 519 | 610 | 632 | 784 | 829 | 14 | 443 | 565 | 9 |
|---|---|---|---|---|---|---|---|---|---|---|---|---|---|---|---|---|---|---|---|---|
| Age | 66 | 55 | 66 | 49 | 66 | 61 | 70 | 65 | 53 | 55 | 57 | 61 | 68 | 57 | 44 | 33 | 33 | 60 | 62 | 68 |
| *PPM1D* | | | | | | | | | | | | | | | | | | | | |
| *TP53* | | | | | | | | | | | | | | | | | | | | |
| *ATM* | | | | | | | | | | | | | | | | | | | | |
| *SRCAP* | | | | | | | | | | | | | | | | | | | | |
| *BRCC3* | | | | | | | | | | | | | | | | | | | | |
| *DNMT3A* | 3 | 1 | 1 | 2 | 1 | 1 | 1 | 1 | 1 | 1 | 1 | 1 | 2 | 1 | 1 | 1 | 1 | 1 | 2 | 1 |
| *TET2* | | 2 | 4 | 1 | 1 | | | 1 | | | | | | | | | | | | |
| *ZNF318* | 1 | | | | | 1 | | | | | | | | | | | | | | |
| *ASXL1* | | | | | | | | | | | | | | | | | | | | |
| *BCORL1* | | | | | | | | | | | | | | | | | | | | |
| *GNAS* | | | | | | | | | | | | | | | | | | | | |
| *SMC1A* | | | | | | | | | | | | | | | | | | | | 1 |
| *STAT3* | | | | 1 | | | | | | | | | | | | | | | | |
| *CUX1* | | | | | | 1 | | | | | | | | | | | | | | |
| *CBL* | | | | | | | | | | | | | | | | | | | | |
| *KRAS* | | | | | | | | | | | | | | | | | | | | |
| *BCOR* | | | | | | | | | | | | | | | | | | | | |
| *EZH2* | | | | | | | | | | | | | | | | | | | | |
| *GNB1* | | | | | | | 1 | | | | | | | | | | | | | |
| *SMC3* | | | | | | | | | | | | | | | | | | | | |
| *FLT3* | | | | | | | | | | | | | | | | | | | | |
| *CSF3R* | | | | | | | | | | | | | | | | | | | | |
| *IDH2* | | | | | | | | | | | | | | | | | | | | |
| *ZRSR2* | | | | | | | | | | | | | | | | | | | | |
| *SF1* | | | | | | | | | | | | | | | | | | | | |
| *JAK2* | | | | | | | | | | | | | | | | | | | | |
| *SETD2* | | | | | | | | | | | | | | | | | | | | |
| *RUNX1* | | | | | | | | | | | | | | | | | | | | |
| *SRSF2* | | | | | | | | | | | | | | | | | | | | |
| *ZBTB33* | | | | | | | | | | | | | | | | | | | | |
| *CREBBP* | | | | | | | | | | | | | | | | | | | | |
| *KDM6A* | | | | | | | | | | | | | | | | | | | | |
| *MYD88* | | | | | | | | | | | | | | | | | | | | |
| *PHF6* | | | | | | | | | | | | | | | | | | | | |
| *PTPN11* | | | | | | | | | | | | | | | | | | | | |
| *STAG2* | | | | | | | | | | | | | | | | | | 1 | | |
| *SETBP1* | | | | | | | | | | | | | | | | | | | | |
| Total | 4 | 3 | 5 | 4 | 2 | 3 | 2 | 2 | 1 | 1 | 1 | 1 | 2 | 1 | 1 | 1 | 1 | 2 | 2 | 2 |

| Patient | 263 | 854 | 20 | 192 | 137 | 654 | 880 | 900 | 35 | 66 | 104 | 106 | 473 | 611 | 673 | 734 | 765 | 776 | 789 | 791 | 339 |
|---|---|---|---|---|---|---|---|---|---|---|---|---|---|---|---|---|---|---|---|---|---|
| Age | 39 | 66 | 59 | 65 | 57 | 71 | 56 | 42 | 55 | 41 | 64 | 43 | 25 | 36 | 40 | 30 | 18 | 40 | 27 | 32 | 26 |
| *PPM1D* | | | | | | | | | | | | | | | | | | | | | |
| *TP53* | | | | | | | | | | | | | | | | | | | | | |
| *ATM* | | | | | | | | | | | | | | | | | | | | | |
| *SRCAP* | | | | | | | | | | | | | | | | | | | | | |
| *BRCC3* | | | | | | | | | | | | | | | | | | | | | |
| *DNMT3A* | 1 | | | | | | | | | | | | | | | | | | | | |
| *TET2* | | 2 | 1 | 1 | | | | | | | | | | | | | | | | | |
| *ZNF318* | | | | | 1 | | | | | | | | | | | | | | | | |
| *ASXL1* | | | | | | | | | | | | | | | | | | | | | |
| *BCORL1* | | | | | | 1 | 1 | | | | | | | | | | | | | | |
| *GNAS* | | 1 | | | | | | | | | | | | | | | | | | | |
| *SMC1A* | | | | | | | | | | | | | | | | | | | | | |
| *STAT3* | | | | | | | | | | | | | | | | | | | | | |
| *CUX1* | | | | | | | | | | | | | | | | | | | | | |
| *CBL* | | | | | | | | | | | | | | | | | | | | | |
| *KRAS* | | | | | | | | | | | | | | | | | | | | | |
| *BCOR* | | | | | | | | | | | | | | | | | | | | | |
| *EZH2* | | | | | | | | | | | | | | | | | | | | | |
| *GNB1* | | | | | | | | | | | | | | | | | | | | | |
| *SMC3* | | | | | | | | | | | | | | | | | | | | | |
| *FLT3* | | | | | | | | | | | | | | | | | | | | | |
| *CSF3R* | | | | | | | | | | | | | | | | | | | | | |
| *IDH2* | | | | | | | | | | | | | | | | | | | | | |
| *ZRSR2* | | | | | | | | | | | | | | | | | | | | | |
| *SF1* | | | | | | | | | | | | | | | | | | | | | |
| *JAK2* | | | | | | | | | | | | | | | | | | | | | |
| *SETD2* | | | | | | | | | | | | | | | | | | | | | |
| *RUNX1* | | | | | | | | | | | | | | | | | | | | | |
| *SRSF2* | | 1 | | | | | | | | | | | | | | | | | | | |
| *ZBTB33* | | | | | | | | | | | | | | | | | | | | | |
| *CREBBP* | | | | | | | | | | | | | | | | | | | | | |
| *KDM6A* | | | | | | | | | | | | | | | | | | | | | |
| *MYD88* | | | | | | | | | | | | | | | | | | | | | |
| *PHF6* | | | | | | | | | | | | | | | | | | | | | |
| *PTPN11* | | | | | | | | | | | | | | | | | | | | | |
| *STAG2* | | | | | | | | | | | | | | | | | | | | | |
| *SETBP1* | | | | | | | | | | | | | | | | | | | | | |
| Total | 1 | 4 | 1 | 1 | 1 | 1 | 1 | 0 | 0 | 0 | 0 | 0 | 0 | 0 | 0 | 0 | 0 | 0 | 0 | 0 | 0 |

## Malignancy without cytotoxic therapy exposure

| Patient | 415 | 908 | 350 | 546 | 911 | 405 | 559 | 975 | 757 | 512 | 1030 | 511 | 760 | 793 | 1002 | 171 | 794 | 798 | 731 |
|---|---|---|---|---|---|---|---|---|---|---|---|---|---|---|---|---|---|---|---|
| Age | 72 | 72 | 72 | 64 | 67 | 66 | 57 | 74 | 67 | 65 | 64 | 59 | 60 | 61 | 67 | 60 | 71 | 68 | 69 |
| *PPM1D* | | | | | | | | | | | | | | | | | | | |
| *TP53* | 1 | 1 | | | | | | | | | | | | | | | | | |
| *ATM* | | | | | | | | | | | | | | | | | | | |
| *SRCAP* | | | 1 | 1 | | | | | | | | | | | | | | | |
| *BRCC3* | | | | | 1 | | | | | | | | | | | | | | |
| *DNMT3A* | 1 | | 1 | 1 | | 3 | 2 | 2 | 1 | 1 | 1 | 1 | 1 | 1 | 2 | 1 | | | |
| *TET2* | | | 1 | | | | 2 | | 1 | | | | | | | | 4 | 1 | 2 |
| *ZNF318* | | 1 | 1 | | | | | | | | | | | | | | | | |
| *ASXL1* | 1 | | | | | | | | | | | | | 1 | | | 1 | | |
| *BCORL1* | | | | | | | | | 1 | | | | | | | | | | |
| *GNAS* | | | | | | | | | | 1 | | | | | | | | | |
| *SMC1A* | | | | | | | | | | | | | | | | | | | |
| *STAT3* | | | 1 | | | | | | | | | | | | | | | | |
| *CUX1* | | | | | | | | | | | | | | | | | | 1 | |
| *CBL* | | | | | 2 | | | | | | | | | | | | | | 1 |
| *KRAS* | | | | | | | | | | | | | | | | | | | |
| *BCOR* | | | | | | | | | | | | | | | | | | | |
| *EZH2* | | | | | | | | | | | | | | | | | | | |
| *GNB1* | | | | | | | | | | | | | | | | | | | |
| *SMC3* | | | | | | | | | | | | | | | | | | | |
| *FLT3* | | | | | | | | | | | | | | | | | | | |
| *CSF3R* | | | | | | | | | | | | | | | | | | | |
| *IDH2* | | | | | | | | | | | | | | | | | | 1 | |
| *ZRSR2* | | | | | | | | | | | | | | | | | | | |
| *SF1* | | | | | | | | | | | | | | | | | | | |
| *JAK2* | | | | | | | | | | | | | | | | | | | |
| *SETD2* | | | | | | | | | | | | | | | | | | | |
| *RUNX1* | | | | | | | | | | | | | | | | | | | |
| *SRSF2* | | | | | | | | | | | | | | | | | | | |
| *ZBTB33* | | | 1 | | | | | | | | 1 | | | | | | | | |
| *CREBBP* | | | | | | | | | | | | | | | | | | | |
| *KDM6A* | | | | 2 | | | | | | | | | | | | | | | |
| *MYD88* | | | | | | | | | | | | | | | | | | | |
| *PHF6* | | | | | | | | | | 1 | | | | | | | | | |
| *PTPN11* | | | 1 | | | | | | | | | | | | | | | | |
| *STAG2* | | | | | | | | | | | | | | | | | | | |
| *SETBP1* | | | | | | | | | | | | | | | | | | | |
| Total | 3 | 2 | 7 | 4 | 3 | 3 | 4 | 2 | 3 | 3 | 2 | 1 | 1 | 2 | 2 | 1 | 5 | 3 | 3 |

| Patient | 827 | 879 | 782 | 665 | 855 | 103 | 18 | 27 | 76 | 120 | 573 | 602 | 609 | 656 | 684 | 700 | 730 | 797 | 931 |
|---|---|---|---|---|---|---|---|---|---|---|---|---|---|---|---|---|---|---|---|
| Age | 60 | 64 | 66 | 47 | 51 | 45 | 47 | 39 | 43 | 45 | 67 | 57 | 39 | 51 | 71 | 64 | 55 | 62 | 54 |
| *PPM1D* | | | | | | | | | | | | | | | | | | | |
| *TP53* | | | | | | | | | | | | | | | | | | | |
| *ATM* | | | | | | | | | | | | | | | | | | | |
| *SRCAP* | | | | | | | | | | | | | | | | | | | |
| *BRCC3* | | | | | | | | | | | | | | | | | | | |
| *DNMT3A* | | | | | | | | | | | | | | | | | | | |
| *TET2* | 1 | 1 | | | | | | | | | | | | | | | | | |
| *ZNF318* | | | 1 | | | | | | | | | | | | | | | | |
| *ASXL1* | | | | | | | | | | | | | | | | | | | |
| *BCORL1* | | | | | | | | | | | | | | | | | | | |
| *GNAS* | | | | | | | | | | | | | | | | | | | |
| *SMC1A* | | | 1 | | | | | | | | | | | | | | | | |
| *STAT3* | | | | 1 | | | | | | | | | | | | | | | |
| *CUX1* | | | | | | | | | | | | | | | | | | | |
| *CBL* | | 1 | | | | | | | | | | | | | | | | | |
| *KRAS* | | | | | 1 | | | | | | | | | | | | | | |
| *BCOR* | | | | | | | | | | | | | | | | | | | |
| *EZH2* | | | | | | | | | | | | | | | | | | | |
| *GNB1* | | | | | | | | | | | | | | | | | | | |
| *SMC3* | | | | | | | | | | | | | | | | | | | |
| *FLT3* | | | | | | | | | | | | | | | | | | | |
| *CSF3R* | | | 1 | | | | | | | | | | | | | | | | |
| *IDH2* | | | | | | | | | | | | | | | | | | | |
| *ZRSR2* | | | | | | | | | | | | | | | | | | | |
| *SF1* | | | | | | | | | | | | | | | | | | | |
| *JAK2* | | | | | | | | | | | | | | | | | | | |
| *SETD2* | | | | | | | | | | | | | | | | | | | |
| *RUNX1* | | | | | | | | | | | | | | | | | | | |
| *SRSF2* | | | | | | | | | | | | | | | | | | | |
| *ZBTB33* | | | | | | | | | | | | | | | | | | | |
| *CREBBP* | | | | | | | | | | | | | | | | | | | |
| *KDM6A* | | | | | | | | | | | | | | | | | | | |
| *MYD88* | | | | | | | | | | | | | | | | | | | |
| *PHF6* | | | | | | | | | | | | | | | | | | | |
| *PTPN11* | | | | | | | | | | | | | | | | | | | |
| *STAG2* | | | | | | | | | | | | | | | | | | | |
| *SETBP1* | | | | | | | | | | | | | | | | | | | |
| Total | 1 | 2 | 3 | 1 | 1 | 0 | 0 | 0 | 0 | 0 | 0 | 0 | 0 | 0 | 0 | 0 | 0 | 0 | 0 |

## Healthy donor

| Patient | D-1103 | D-990 | D-816 | D-1185 | D-1212 | D-838 | D-1100 | D-953 | D-1043 | D-810 | D-993 | D-858 | D-909 | D-929 | D-937 | D-938 | D-954 | D-1002 | D-1096 |
|---|---|---|---|---|---|---|---|---|---|---|---|---|---|---|---|---|---|---|---|
| Age | 64 | 76 | 67 | 62 | 66 | 60 | 31 | 74 | 57 | 55 | 66 | 52 | 63 | 72 | 53 | 41 | 21 | 50 | 30 |
| *PPM1D* | 1 | | | | | | | | | | | | | | | | | | |
| *TP53* | | 2 | 1 | | | | | | | | | | | | | | | | |
| *ATM* | | | | | | | | | | | | | | | | | | | |
| *SRCAP* | | | | 2 | | | | | | | | | | | | | | | |
| *BRCC3* | | | | | | | | | | | | | | | | | | | |
| *DNMT3A* | | | | 2 | 2 | 1 | 1 | 3 | 1 | | | | | | | | | | |
| *TET2* | | 3 | 1 | | 2 | | | | | | | | | | | | | | |
| *ZNF318* | | | | | | | | | | | | | | | | | | | |
| *ASXL1* | | | | | | | | 1 | | | | | | | | | | | |
| *BCORL1* | | | | | | | | | | | | | | | | | | | |
| *GNAS* | | | | | | | | | | | | | | | | | | | |
| *SMC1A* | | | | | | | | | | | | | | | | | | | |
| *STAT3* | | | | | | | | | | | | | | | | | | | |
| *CUX1* | | | | | | | | | | | | | | | | | | | |
| *CBL* | | | | | | | | | | | | | | | | | | | |
| *KRAS* | | | | | | | | | | | | | | | | | | | |
| *BCOR* | | | | | | | | | | 2 | | | | | | | | | |
| *EZH2* | | | | | | | | | | | | | | | | | | | |
| *GNB1* | | | | | | | | | | | | | | | | | | | |
| *SMC3* | | | | | | | | | | | | | | | | | | | |
| *FLT3* | | | | | | | | | | | | | | | | | | | |
| *CSF3R* | | | | | | | | | | | | | | | | | | | |
| *IDH2* | | | | | | | | | | | | | | | | | | | |
| *ZRSR2* | | | | | | | | | | | | | | | | | | | |
| *SF1* | | | | | | | | | | | | | | | | | | | |
| *JAK2* | | | | | | | | | | | | | | | | | | | |
| *SETD2* | | | | | | | | | | | 1 | | | | | | | | |
| *RUNX1* | | | | | | | | | | | | | | | | | | | |
| *SRSF2* | | | | | | | | | | | | | | | | | | | |
| *ZBTB33* | | | | | | | | | | | | | | | | | | | |
| *CREBBP* | | | | | | | | | | | | | | | | | | | |
| *KDM6A* | | | | | | | | | | | | | | | | | | | |
| *MYD88* | | | 2 | | | | | | | | | | | | | | | | |
| *PHF6* | | | | | | | | | | | | | | | | | | | |
| *PTPN11* | | | | | | | | | | | | | | | | | | | |
| *STAG2* | | | | | | | | | | | | | | | | | | | |
| *SETBP1* | | | 1 | | | | | | | | | | | | | | | | |
| Total | 1 | 5 | 5 | 4 | 4 | 1 | 1 | 4 | 1 | 2 | 1 | 0 | 0 | 0 | 0 | 0 | 0 | 0 | 0 |

**Supplementary Figure 4. Multiple variants are often present in individual patients following cytotoxic therapy.** Highlighted are the gene-specific variants detected in pheresis samples with the total number of variants noted. Multiple variants are often present in individual patients after cytotoxic therapy, including multiple variants in the same gene (e.g. in *PPM1D*, *TP53*, etc.). Green: lymphoma patients. Blue: myeloma patients. Gray: healthy donors. Patients with expanded clones carrying mutations in DNA damage response genes (i.e. *PPM1D*, *TP53*, *ATM*, *SRCAP*, and *BRCC3*) are on the left.

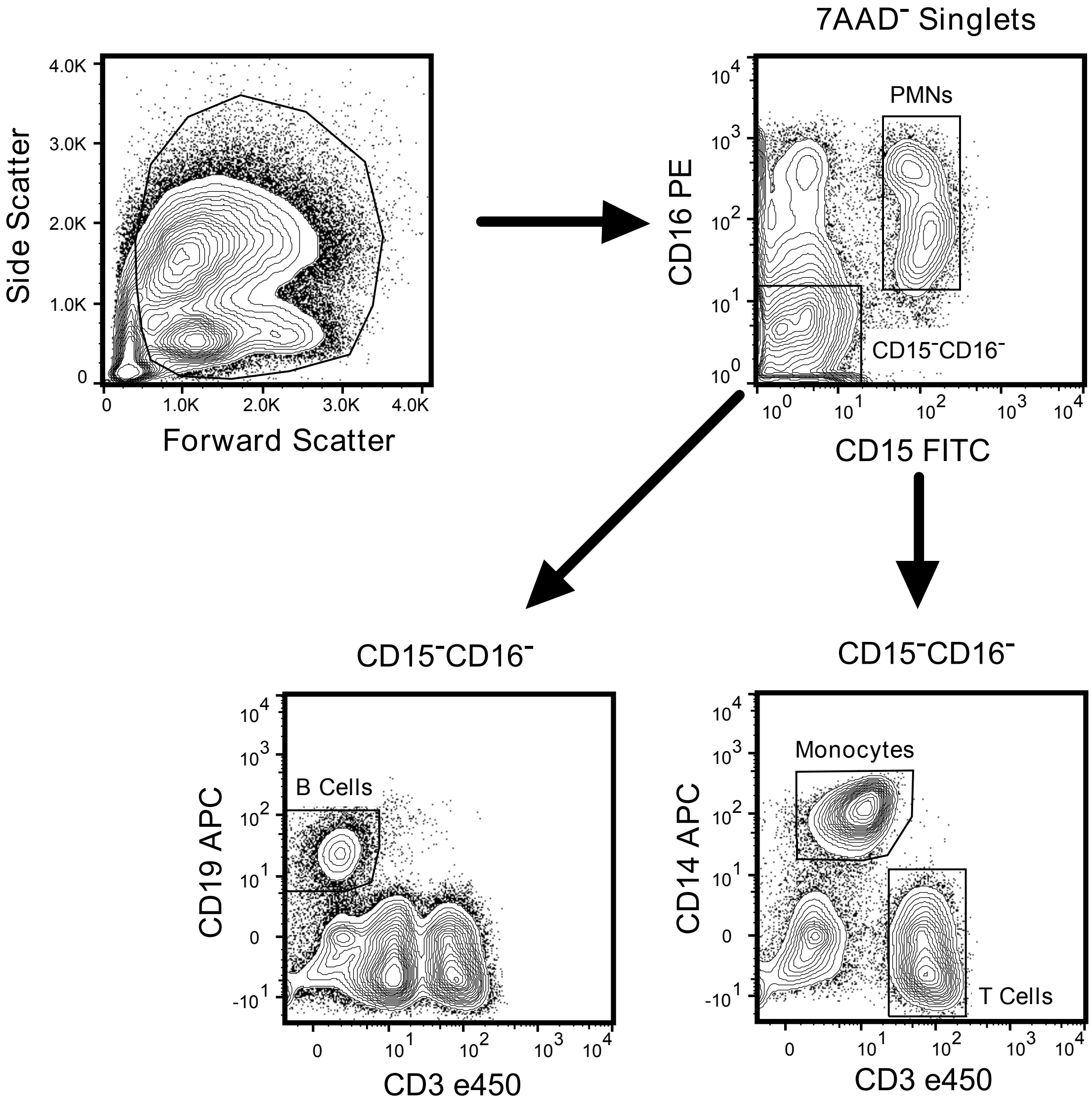


**Supplementary Figure 5. Representative sorting strategy to isolate monocytes, neutrophils, B cells, and T cells.** Monocytes were defined as $CD15^-CD16^-CD14^+$ cells. Neutrophils (PMNs) were defined as $CD15^+CD16^+$ cells. T cells were defined as $CD15^-CD16^-CD3^+$ cells. B cells were defined as $CD15^-CD16^-CD19^+$ cells. Due to most patients receiving rituximab, B cells were only able to be isolated from one patient.

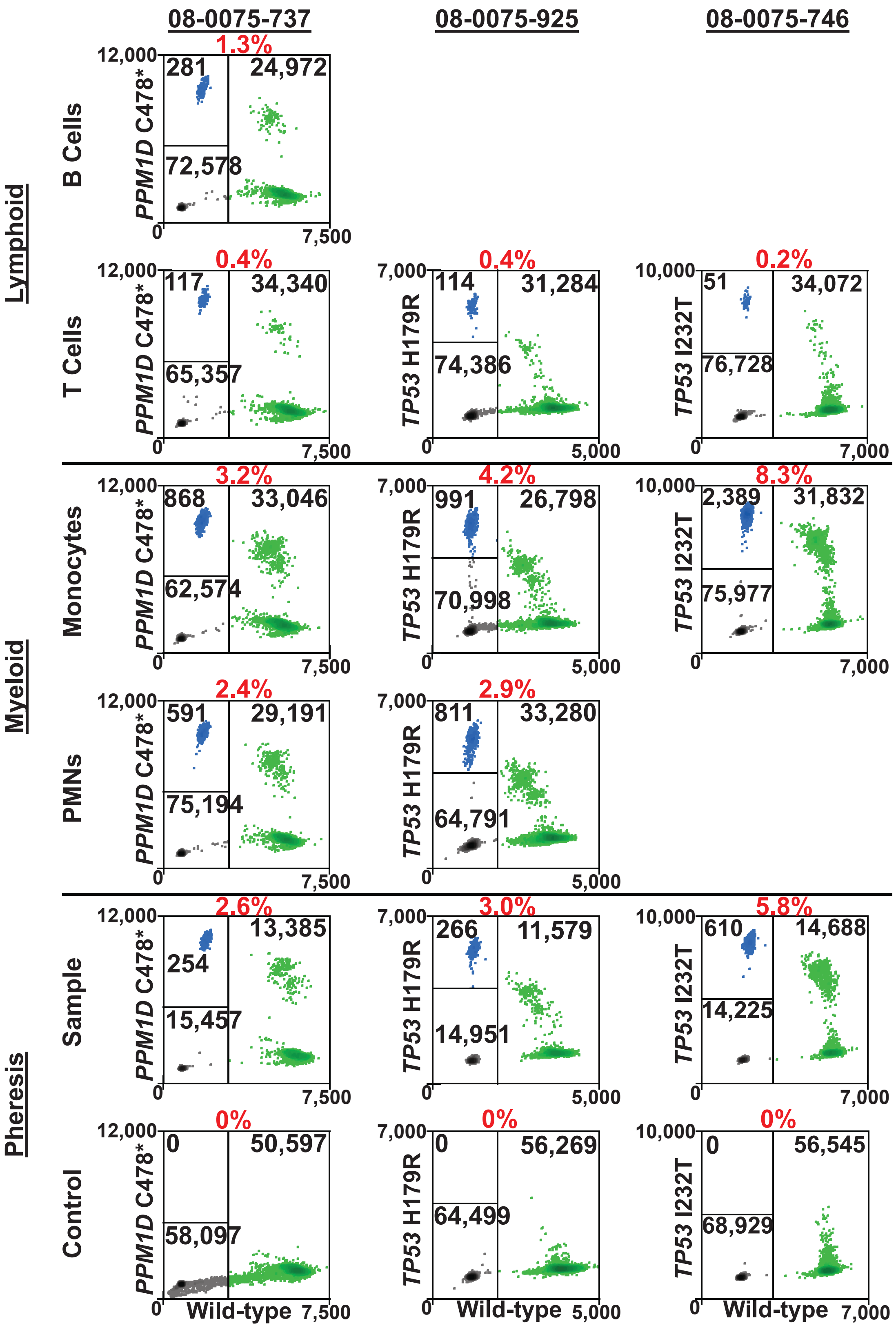


**Supplementary Figure 6. Representative droplet digital PCR to determine the VAFs of variants of interest in sorted cell populations.** Droplets containing only the mutant allele are highlighted in blue; droplets containing the wild-type allele (with or without the mutant allele) are highlighted in green; empty droplets are gray. The number of droplets in each gate is indicated. VAFs were determined using Poisson statistics and highlighted in red. PMNs: neutrophils.

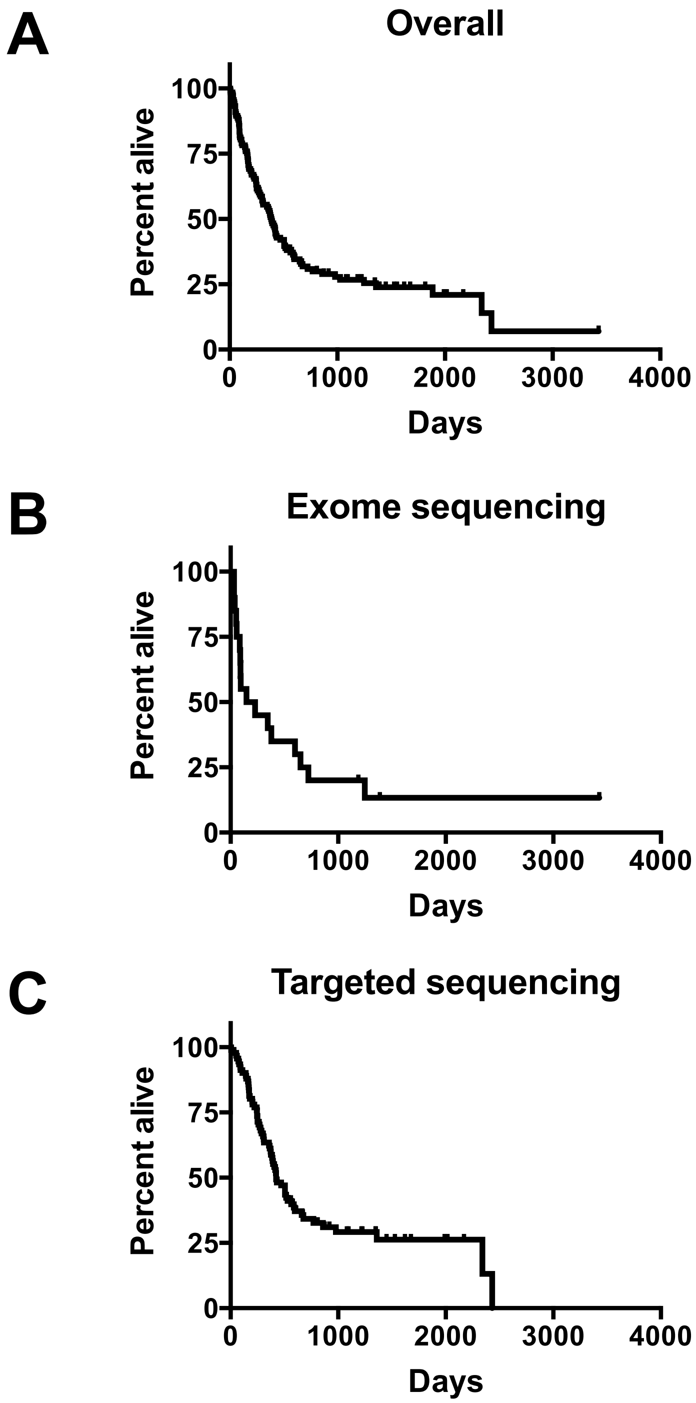


**Supplementary Figure 7. Overall survival for 134 t-AML/t-MDS patients.** 20 patients had exome sequencing with one published previously. 92 patients had targeted sequencing with 19 previously reported on using a different gene targeting panel. 22 patients had previously published whole genome sequencing. **a,** Overall t-AML/t-MDS cohort (n=134). **b,** Exome sequencing cohort (n=20). **c,** Targeted sequencing cohort (n=92).

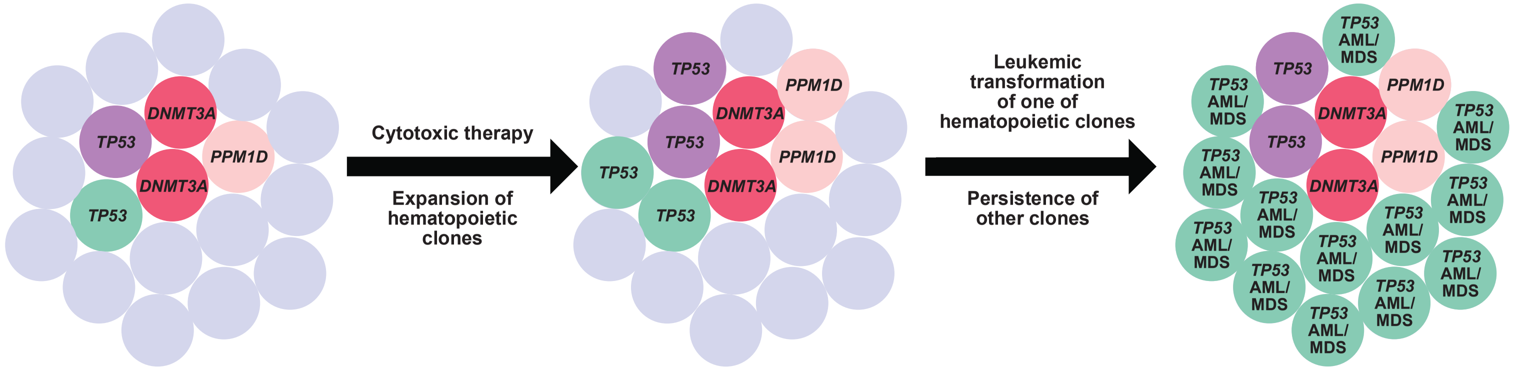


**Supplementary Figure 8. Model of how cytotoxic therapy shapes hematopoietic clonal evolution.** Cytotoxic therapy provides a fitness advantage to multiple hematopoietic clones harboring specific mutations (e.g. in *TP53*, *PPM1D*, etc.), allowing them to expand. These clones have differing leukemogenic potential with certain clones (particularly those with mutations in *TP53*) more likely to progress to AML/MDS than others. However, non-leukemic expanded clones are often long-lived and may co-exist with the malignant clone at diagnosis.

**Supplementary Table 1** Detailed clinical data for myeloma patients assessed with error-corrected sequencing

| Patient (UPN) | Gender | Age | Myeloma treatment prior to pheresis collection | Radiation? (Yes/No) | Cytotoxic therapy? (Yes/No) | Time from initial treatment to transplant (months) | Conditioning regimen |
|---|---|---|---|---|---|---|---|
| 14 | M | 33 | Thalidomide/dexamethasone x 6 | Yes | Yes | 6 | Melphalan |
| 18 | F | 47 | Thalidomide/dexamethasone | No | No | 27 | Melphalan |
| 27 | F | 39 | Thalidomide/dexamethasone | No | No | 5 | Melphalan |
| 76 | M | 43 | Thalidomide/dexamethasone x 7 months; lenalidamide/dexamethasone x 2 | No | No | 16 | Melphalan |
| 103 | M | 45 | Thalidomide/dexamethasone; lenalidamide/dexamethasone | No | No | 14 | Melphalan |
| 120 | F | 45 | Thalidomide/dexamethasone | No | No | 5 | Melphalan |
| 339 | M | 26 | VRD x 4 | Yes | Yes | 27 | Melphalan |
| 350 | F | 72 | VRD x 3 | No | No | 4 | Melphalan |
| 405 | F | 66 | VD x 3 | No | No | 4 | Melphalan |
| 415 | M | 72 | VRD x 3 | No | No | 4 | Melphalan |
| 443 | M | 60 | Doxil/dexamethasone/bortezomib x 3 | No | Yes | 5 | Melphalan |
| 457 | M | 68 | RD; VRD | Yes | Yes | N.A. | N.A. |
| 511 | M | 59 | Thalidomide/dexamethasone | No | No | 26 | Melphalan |
| 512 | F | 65 | KRd x 5 | No | No | unknown | Melphalan |
| 546 | F | 64 | VD x 4 | No | No | 5 | Melphalan |
| 559 | F | 57 | VRD x 4 | No | No | 6 | Melphalan |
| 565 | F | 62 | VD x 1; VRD x 3 | Yes | Yes | 3 | Melphalan |
| 570 | F | 59 | VD x 8 | Yes | Yes | 19 | Melphalan |
| 573 | M | 67 | VRD x 3 | No | No | 4 | Melphalan |
| 599 | M | 76 | VD x 7; RD x 4; VD x 4; DVD x 2 | Yes | Yes | 21 | Melphalan |
| 601 | F | 72 | RD x 7 | Yes | Yes | unknown | Melphalan |
| 602 | F | 57 | VRD x 4 | No | No | 4 | Melphalan |
| 609 | M | 39 | VRD x 4 | No | No | 5 | Melphalan |
| 656 | M | 51 | VRD x 4 | No | No | 5 | Melphalan |
| 665 | F | 47 | VD x 4; lenalidamide x 3 | No | No | 4 | Melphalan |
| 684 | F | 71 | RD; VRD x 3; cyclophosphamide mobilization | No | No | 12 | Melphalan |
| 700 | M | 64 | VD x 1; VRD < 1; VD x 3 | No | No | 6 | Melphalan |
| 730 | M | 55 | VD x 4 | No | No | 3 | Melphalan |
| 731 | M | 69 | VRD x 3 | No | No | 4 | Melphalan |
| 739 | M | 66 | VRD x 3 | Yes | Yes | 5 | Melphalan |
| 757 | M | 67 | VD x 5 | No | No | unknown | Melphalan |
| 760 | F | 60 | VRD x 4 | No | No | 5 | Melphalan |
| 782 | M | 66 | VRD x 3 | No | No | 4 | Melphalan |
| 793 | M | 61 | VRD x 3 | No | No | 3 | Melphalan |
| 794 | M | 71 | VRD x 3 | No | No | 4 | Melphalan |
| 797 | F | 62 | VD x 1; VRD x 4 | No | No | 5 | Melphalan |
| 798 | M | 68 | VRD x 4 | No | No | 7 | Melphalan |
| 827 | M | 60 | VD x 4 | No | No | 5 | Melphalan |
| 855 | M | 51 | VRD x 4 | No | No | 4 | Melphalan |
| 879 | M | 64 | KRd x 4 | No | No | 4 | Melphalan |
| 907 | M | 41 | KRd x 4 | Yes | Yes | 6 | Melphalan |
| 908 | M | 72 | VRD x 4 | No | No | 5 | Melphalan |
| 911 | F | 67 | VRD x 4 | No | No | 4 | Melphalan |
| 931 | M | 54 | KRd x 3 | No | No | 4 | Melphalan |
| 954 | F | 66 | VD x 1; VRD x 3 | Yes | Yes | 5 | Melphalan |
| 968 | M | 55 | VRD x 4 | Yes | Yes | 4 | Melphalan |
| 975 | F | 74 | VRD x 3; RD x 1 | No | No | 8 | Melphalan |
| 987 | M | 72 | VD x 2; VRD x 5 | Yes | Yes | 6 | Melphalan |
| 1002 | M | 67 | VRD x 4 | No | No | 4 | Melphalan |
| 1030 | F | 64 | VD x 6 | No | No | 9 | Melphalan |

N.A.: not applicable; UPN: unidentified patient number
Treatment regimens are as follows:
DVD: doxil, vincristine, dexamethasone
KRd:  carfilzomib, lenalidamide, dexamethasone
RD: lenalidamide, dexamethasone
VD: bortezomib, dexamethasone
VRD: bortezomib, lenalidamide, dexamethasone

**Supplementary Table 2** Genes targeted by error-corrected sequencing

| | | | |
|---|---|---|---|
| *ASXL1* | *EZH2* | *NRAS* | *SRCAP* |
| *ATM* | *FLT3* | *PHF6* | *SRSF2* |
| *ATRX* | *GNAS* | *PPM1D* | *STAG2* |
| *BCOR* | *GNB1* | *PTPN11* | *STAT3* |
| *BCORL1* | *IDH1* | *RAD21* | *TET2* |
| *BRCC3* | *IDH2* | *RUNX1* | *TP53* |
| *CBL* | *JAK2* | *SETBP1* | *U2AF1* |
| *CEBPA* | *KDM6A* | *SETD2* | *ZBTB33* |
| *CREBBP* | *KRAS* | *SF1* | *ZNF318* |
| *CSF3R* | *LUC7L2* | *SF3B1* | *ZRSR2* |
| *CUX1* | *MYD88* | *SMC1A* | |
| *DNMT3A* | *NPM1* | *SMC3* | |

**Supplementary Table 3** Association between patient clinical features and hematopoietic clonal expansion

| | Age | | Radiation therapy | | Radiation therapy (age-adjusted) | | Chemotherapy | | Chemotherapy (age-adjusted) | | Cytoreductive therapy | | Cytoreductive therapy (age-adjusted) | |
|---|---|---|---|---|---|---|---|---|---|---|---|---|---|---|
| | **IRR** | **P Value** | **IRR** | **P Value** | **IRR** | **P Value** | **IRR** | **P Value** | **IRR** | **P Value** | **IRR** | **P Value** | **IRR** | **P Value** |
| **Number of mutations** | 1.05 | < 0.001 | 1.10 | 0.70 | 1.18 | 0.45 | 1.51 | 0.03 | 1.85 | < 0.001 | 1.84 | 0.001 | 2.28 | < 0.001 |
| **Presence of:** | **OR** | **P Value** | **OR** | **P Value** | **OR** | **P Value** | **OR** | **P Value** | **OR** | **P Value** | **OR** | **P Value** | **OR** | **P Value** |
| **Any Mutation** | 1.10 | < 0.001 | 2.19 | 0.25 | 3.98 | 0.07 | 2.23 | 0.06 | 4.87 | 0.003 | 2.98 | 0.009 | 9.21 | < 0.001 |
| ***DNMT3A*** | 1.04 | 0.003 | 1.95 | 0.19 | 2.20 | 0.13 | 1.13 | 0.85 | 1.32 | 0.54 | 1.89 | 0.10 | 2.34 | 0.03 |
| ***PPM1D*** | 1.03 | 0.26 | 0.91 | 1.00 | 0.95 | 1.00 | 19.54 | <0.001 | 22.80 | < 0.001 | 13.61 | 0.002 | 15.45 | < 0.001 |
| ***TP53*** | 1.09 | < 0.001 | 1.93 | 0.35 | 2.17 | 0.29 | 2.80 | 0.07 | 4.13 | 0.01 | 3.49 | 0.04 | 4.94 | 0.009 |
| ***SRCAP*** | 1.02 | 0.32 | 0.26 | 0.30 | 0.27 | 0.32 | 3.28 | 0.07 | 3.63 | 0.05 | 3.41 | 0.09 | 3.76 | 0.06 |
| ***TET2*** | 1.08 | < 0.001 | 0.29 | 0.14 | 0.28 | 0.15 | 1.27 | 0.73 | 1.65 | 0.37 | 1.03 | 1.00 | 1.28 | 0.76 |
| ***ZNF318*** | 1.09 | 0.004 | 1.09 | 1.00 | 1.14 | 1.00 | 1.52 | 0.63 | 2.04 | 0.33 | 3.11 | 0.13 | 4.21 | 0.05 |
| ***ATM*** | 1.02 | 0.69 | 0.86 | 1.00 | 0.89 | 1.00 | 8.47 | 0.03 | 9.05 | 0.03 | 6.07 | 0.08 | 6.50 | 0.06 |

IRR: Incident rate ratio; OR: exact odds ratio

**Supplementary Table 4** Gene panel for targeted t-AML/t-MDS sequencing

| | | | | | | |
|---|---|---|---|---|---|---|
| *ABCG2* | *CMYA5* | *FAM40B* | *IKZF4* | *MLL3* | ***PPM1D*** | *ST13P13* |
| *ABTB1* | *CNTN5* | *FAM47A* | *ILDR1* | *MROH5* | *PPP1R3A* | ***STAG2*** |
| *ADAM11* | *CNTNAP4* | *FAM57B* | *ITPR3* | *MTA2* | *PRAMEF2* | ***STAT3*** |
| *ADCY5* | *COL12A1* | *FAM5C* | *JAK1* | *MTMR8* | *PRPF4B* | *STC2* |
| *AKAP13* | *COL5A3* | *FAM65A* | ***JAK2*** | *MTUS2* | *PRPF8* | *STRN* |
| *ALPK3* | ***CREBBP*** | *FAM70B* | *JAK3* | *MUC16* | *PSME4* | *SUZ12* |
| *ANK2* | *CRISPLD1* | *FCGBP* | *KCNA4* | *MUC5B* | *PTCH1* | *SYNGAP1* |
| *APOB* | *CROCC* | *FKBP8* | *KCNH2* | *MYC* | ***PTPN11*** | *SYT15* |
| *ARAP2* | *CSMD1* | *FLG* | *KCNK13* | *MYH4* | *PTPRN* | *TCEAL3* |
| ***ASXL1*** | *CSMD3* | *FLRT2* | *KCNQ2* | *MYO5B* | *PTPRT* | *TCEAL6* |
| *ATG16L1* | *CUEDC1* | *FLT1* | *KCNT1* | *MYOC* | ***RAD21*** | *TET1* |
| ***ATM*** | *CUL1* | ***FLT3*** | *KCNU1* | *MYOM3* | *RBBP4* | ***TET2*** |
| *ATP10B* | *DCHS2* | *FOXP1* | *KDM3B* | *NALCN* | *RFC3* | *THRAP3* |
| *ATP2B3* | *DCLK1* | *FREM2* | ***KDM6A*** | *NAV1* | *RIMS1* | *TMEM104* |
| ***BCOR*** | *DDR2* | *FRYL* | *KDR* | *NF1* | *RNF213* | *TNC* |
| ***BCORL1*** | *DDX11* | *GALNTL4* | *KIAA0240* | *NLRC4* | ***RUNX1*** | *TOP3B* |
| *BMPER* | *DDX41* | *GAS6* | *KIAA1267* | *NMUR2* | *RUNX1T1* | ***TP53*** |
| *BOD1L* | *DIS3* | *GATA2* | *KIAA1529* | ***NPM1*** | *RYR1* | *TRA2B* |
| ***BRCC3*** | *DLC1* | *GBP4* | *KIAA1683* | *NR2E1* | *RYR3* | *TRPM3* |
| *BSN* | *DNAH5* | *GIGYF2* | *KIF2B* | ***NRAS*** | *SBF1P1* | *TTBK1* |
| *C10orf118* | *DNAH9* | *GJB3* | *KIT* | *NRXN3* | *SCAF8* | *TUBA3C* |
| *C10orf28* | *DNAI1* | ***GNAS*** | ***KRAS*** | *NTRK3* | *SCARB1* | *TYK2* |
| *C17orf97* | ***DNMT3A*** | ***GNB1*** | *KRT19* | *OR11H12* | *SCML2* | ***U2AF1*** |
| *C5* | *DNMT3B* | *GPR112* | *KRT79* | *OR13H1* | *SCN1A* | *UNC5B* |
| *CACNA1B* | *DOCK2* | *GPR183* | *KSR2* | *OR8B12* | *SEMA3A* | *USP9X* |
| *CACNA1E* | *DRD2* | *GRID1* | *LNX1* | *P2RY2* | *SEMA4A* | *VARS2* |
| *CACNA1G* | *DSCAM* | *GRIK2* | *LOC100129218* | *PCDHA13* | ***SETBP1*** | *VCAN* |
| *CACNA2D3* | *DST* | *GRIK4* | *LOC100132800* | *PCDHA6* | ***SF3B1*** | *WAC* |
| *CADM2* | *DYNC2H1* | *GRM3* | *LOC730032* | *PCDHB1* | *SHC1* | *WT1* |
| *CADPS* | *DYSF* | *GRM8* | *LRBA* | *PCDHB18* | *SHROOM2* | *XIRP1* |
| *CALR* | *E2F8* | *GSTK1* | *LRIT1* | *PDCD2L* | *SI* | ***ZBTB33*** |
| *CBFB* | *EDIL3* | *HECW1* | *LRP1B* | *PHACTR1* | *SLC12A3* | *ZC3H18* |
| ***CBL*** | *EED* | *HIVEP1* | *LRRC4* | ***PHF6*** | *SLC39A5* | ***ZNF318*** |
| *CCDC67* | *EEF1A1P29* | *HMCN1* | *MAGI2* | *PHIP* | ***SMC1A*** | *ZNF687* |
| *CD74* | *EGFR* | *HNRNPK* | *MAP1B* | *PKD1L2* | ***SMC3*** | |
| ***CEBPA*** | *EPHA10* | *HSP90B3P* | *MAP2* | *PKD2L1* | *SMG1* | |
| *CECR2* | *EPPK1* | *HYDIN* | *MED12* | *PKHD1* | *SPEG* | |
| *CELSR3* | *ETV6* | ***IDH1*** | *MEFV* | *PKHD1L1* | *SPEN* | |
| *CHD4* | ***EZH2*** | ***IDH2*** | *MEGF8* | *PLCE1* | ***SRCAP*** | |
| *CLEC18B* | *FAM154B* | *IGHG3* | *MIR142* | *PLEKHH1* | ***SRSF2*** | |

HaloPlex genes highlighted. HaloPlex genes not included in the t-AML/t-MDS targeted list: *ATRX*, *CSF3R*, *CUX1*, *LUC7L2*, *MYD88*, *SETD2*, *SF1*, and *ZRSR2*.
