# Supplementary material for: Cellular stressors contribute to the expansion of hematopoietic clones of varying leukemic potential
Source: Nat Commun. 2018 Jan 31;9:455. doi: 10.1038/s41467-018-02858-0 (PMC5792556; doi:10.1038/s41467-018-02858-0)
Supplement: Supplementary file 3 — Description of Additional Supplementary Files [file 41467_2018_2858_MOESM3_ESM.docx]

**Description of Additional Supplementary Files**

File Name: Supplementary Data 1

Description: Detailed clinical data for lymphoma patients assessed with error-corrected sequencing

File Name: Supplementary Data 2

Description: Variants identified in pheresis or post-transplant samples with error-corrected sequencing

File Name: Supplementary Data 3

Description: Variants identified by error-corrected sequencing in lymphoma patients with paired pre-transplant and post-transplant samples

File Name: Supplementary Data 4

Description: Detailed clinical data for t-AML/t-MDS patients assessed with targeted or exome sequencing

File Name: Supplementary Data 5

Description: Variants identified by exome and targeted sequencing of t-AML/t-MDS
